# Two Cases of Duodenal Ulcers That Developed after Transcatheter Procedures for Unruptured Visceral Artery Aneurysms

**DOI:** 10.1155/2022/9988216

**Published:** 2022-04-07

**Authors:** Masaya Iwamuro, Yusuke Kawai, Mayu Uka, Yusuke Matsui, Takao Hiraki, Yoshiro Kawahara, Hiroyuki Okada

**Affiliations:** ^1^Department of Gastroenterology and Hepatology, Okayama University Graduate School of Medicine, Dentistry, and Pharmaceutical Sciences, Okayama 700-8558, Japan; ^2^Department of Gastroenterology, Mitoyo General Hospital, Kan'onji 769-1695, Japan; ^3^Department of Radiology, Okayama University Graduate School of Medicine, Dentistry, and Pharmaceutical Sciences, Okayama 700-8558, Japan; ^4^Department of Practical Gastrointestinal Endoscopy, Okayama University Hospital, Okayama 700-8558, Japan

## Abstract

Herein, we report two cases of duodenal ulcers that developed after transcatheter procedures for the treatment of unruptured artery aneurysms. Both patients recovered after the administration of nothing by mouth, intravenous fluids, and proton-pump inhibitors. Notably, the duodenal ulcer was unchanged in one patient six days after endovascular treatment and improved in the other patient 13 days after angiography. These cases suggest that conservative treatment is acceptable in patients with duodenal ischemia that develops as an adverse effect of endovascular procedures. The usefulness of esophagogastroduodenoscopy in such patients has also been highlighted.

## 1. Introduction

Aneurysms occurring in the branches of the celiac, superior mesenteric, inferior mesenteric, or renal arteries are called abdominal visceral artery aneurysms [1–4]. Once such aneurysms rupture, mortality markedly increases, even if emergency treatment is performed. Thus, treatment of unruptured aneurysms is recommended depending on the risk factors for rupture, such as aneurysm size, morphology, and location. Currently, elective endovascular embolization is preferred for abdominal visceral artery aneurysms because it is less invasive than open surgical repair. Although endovascular treatment is safely performed in most cases, several procedure-related adverse events have been reported, including bowel ischemia and infarction, distal occlusion, arterial perforation, arterial dissection, and pancreatitis [5]. However, the detailed clinical courses of endovascular treatment-related bowel ischemia and infarction have not been sufficiently reported.

Herein, we report two patients with duodenal ulcers that developed following the transarterial approach for unruptured abdominal visceral artery aneurysms. Both patients spontaneously recovered with conservative treatment.

## 2. Case Presentation

### 2.1. Case 1

A 63-year-old woman underwent treatment for a *Helicobacter pylori* infection. Thereafter, she underwent computed tomography (CT) to investigate persistent epigastric discomfort and was diagnosed with median arcuate ligament syndrome with inferior and superior pancreaticoduodenal artery aneurysms. The patient was prescribed telmisartan, rosuvastatin, and rebamipide for hypertension, hypercholesterolemia, and epigastric symptoms, respectively, and referred to the Department of Radiology of our hospital for further investigation and treatment. Although both endovascular treatment for visceral artery aneurysms and surgical treatment such as release or decompression of the median arcuate ligament have been proposed, endovascular embolization alone was performed as the patient did not wish to undergo surgery.

**Figure 1:**
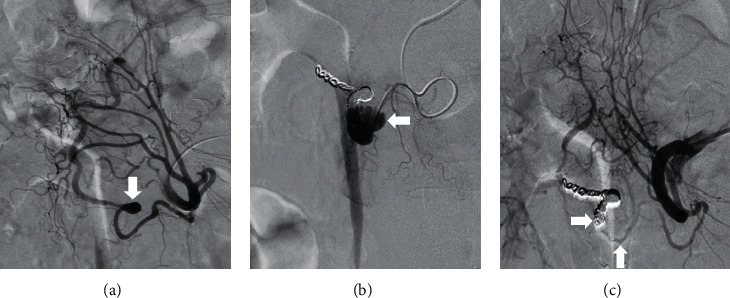
Angiography images of Case 1. A superior pancreaticoduodenal artery aneurysm is seen ((a), arrow). A rupture of the aneurysm after coil embolization of the distal branch ((b), arrow). The artery has been embolized with N-butyl cyanoacrylate ((c), arrows).

**Figure 2:**
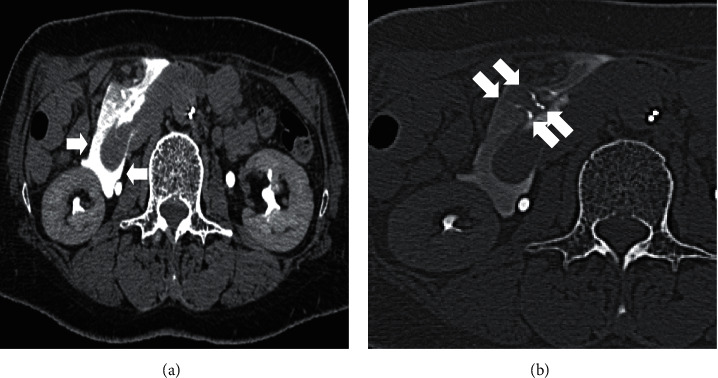
CT images of Case 1 performed immediately after embolization. CT showed leakage of the contrast medium into the retroperitoneum ((a), arrows). Lipiodol retention was observed in the duodenal arteries ((b), arrows). No free air was observed in the abdomen.

**Figure 3:**
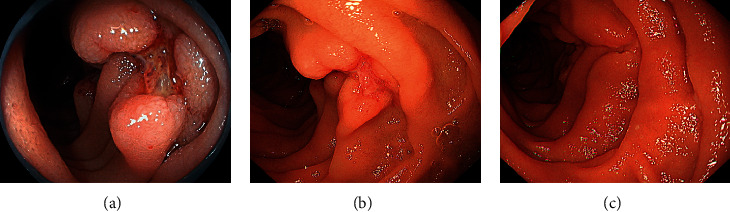
Esophagogastroduodenoscopy images of Case 1. Endoscopy performed three days after endovascular treatment shows multiple ulcers in the second portion of the duodenum (a). The duodenal ulcer is unchanged six days after endovascular treatment (b). Esophagogastroduodenoscopy performed 46 days after aneurysm treatment shows that duodenal ulcers are completely healed (c).

First, treatment of the superior pancreaticoduodenal artery aneurysm was planned (Figure 1(a), arrow). After coil embolization of the distal branch, the aneurysmal sac was filled with coil. However, the proximal side of the aneurysm ruptured (Figure 1(b), arrow) and the patient complained of epigastric pain. The artery was embolized using 33% n-butyl cyanoacrylate diluted with lipiodol (Figure 1(c), arrows). Although CT performed immediately after embolization showed leakage of the contrast medium into the retroperitoneum (Figure 2(a), arrows), no free air was observed in the abdomen. In addition, lipiodol retention was observed in the duodenal arteries (Figure 2(b), arrows). The patient was administered nothing orally, intravenous fluids, or proton-pump inhibitors. After the aneurysm ruptured, the patient developed epigastric pain that persisted for three days but gradually improved. Esophagogastroduodenoscopy performed three days after endovascular treatment revealed multiple ulcers in the second portion of the duodenum (Figure 3(a)). Sucralfate and sodium alginate were additionally prescribed for the duodenal ulcers. Since the duodenal ulcers did not worsen six days after endovascular treatment (Figure 3(b)), she was started on a liquid diet and discharged 10 days after endovascular treatment. Esophagogastroduodenoscopy performed 46 days after endovascular treatment revealed complete healing of duodenal ulcers (Figure 3(c)). Subsequently, the inferior pancreaticoduodenal artery aneurysm was successfully embolized using a coil.

### 2.2. Case 2

**Figure 4:**
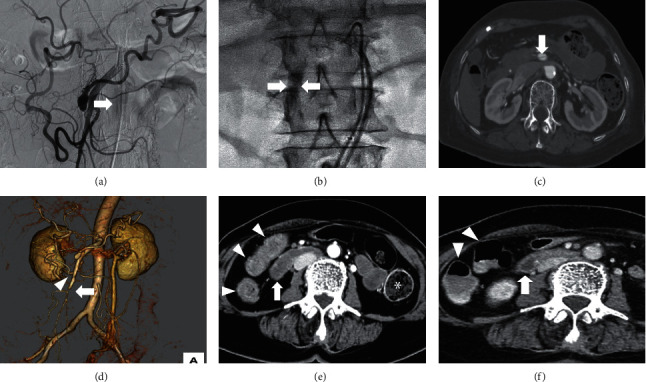
Radiology images of Case 2. A visceral artery aneurism is observed around the uncinate process of pancreas ((a), arrow). During selective cannulation to the aneurysmal neck, retention of the contrast medium is observed along the superior mesenteric artery ((b), arrows). CT shows separation of the inner and outer vascular layers ((c), arrow), confirming the diagnosis of superior mesenteric artery dissection. Stenosis of the roots of the ileocolic ((d), arrow) and right colic arteries ((d), arrowhead) is also observed. In contrast to the intact descending colon ((e), asterisk), mural thickening and delayed, reduced enhancement of the bowel wall are observed in the duodenum ((e), arrow) and ascending colon ((e), arrowhead). CT performed 14 days after angiography revealed improvement in mural thickening of the duodenum ((f), arrow), jejunum, and ascending colon ((f), arrowheads).

An 83-year-old Japanese woman underwent annual abdominal ultrasonography, which revealed a visceral artery aneurysm around the uncinate process of the pancreas. CT showed a dorsal pancreatic artery aneurysm with a diameter of 10 mm and occlusion of the celiac artery. The patient was referred to the Department of Radiology at our hospital for further investigation. The patient was prescribed ursodeoxycholic acid, ethyl icosapentate, and suvorexant for primary biliary cholangitis, hypercholesterolemia, and sleeplessness. She had a history of anterior cervical discectomy and fusion surgery for a cervical disc herniation. We attempted endovascular coiling to block blood flow into the aneurysm and prevent aneurysm rupture (Figure 4(a)).

**Figure 5:**
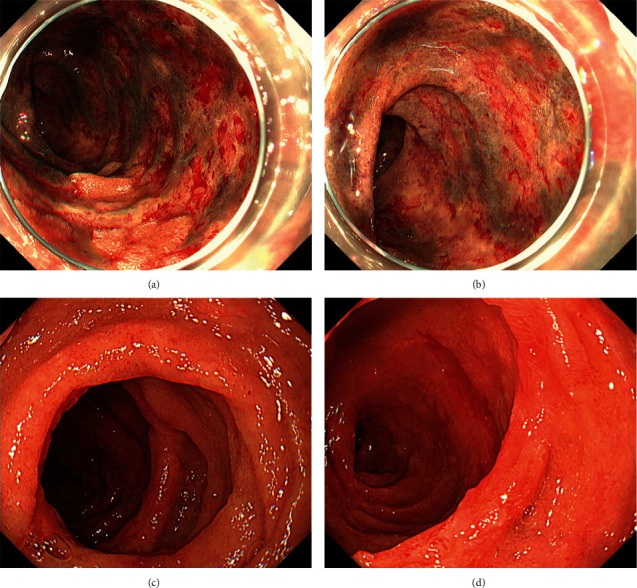
Esophagogastroduodenoscopy images of Case 2. Esophagogastroduodenoscopy performed the day after angiography examination shows extensive exfoliation of the second and third portions of the duodenum (a, b). Thirteen days after angiography, the duodenal mucosal damage has improved (c, d).

During selective cannulation of the aneurysmal neck, retention of contrast medium was observed along the superior mesenteric artery (Figure 4(b)). CT showed separation of the inner and outer vascular layers (Figure 4(c)), confirming the diagnosis of superior mesenteric artery dissection. Stenosis of the roots of the ileocolic (Figure 4(d), arrow) and right colic arteries (Figure 4(d), arrowhead) was also observed. In contrast to the intact descending colon (Figure 4(e), asterisk), the duodenum (Figure 4(e), arrow), jejunum, and ascending colon (Figure 4(e), arrowhead) showed mural thickening and delayed reduced enhancement of the bowel wall. Although the patient complained of abdominal pain and nausea, her symptoms gradually improved. Intravascular embolization was aborted, and the patient was treated with nothing by mouth, intravenous fluids, antibiotics, heparin, and proton-pump inhibitors. As the patient had melena the day after angiography, we performed esophagogastroduodenoscopy, which revealed extensive exfoliation of the second and third portions of the duodenum (Figures 5(a) and 5(b)). The mucosa had turned black. Although there were no features of pancreatitis on computed tomography (CT), the serum amylase level was elevated to 510 U/L. Thus, nafamostat was administered and the patient was treated with total parenteral nutrition. The serum amylase levels gradually normalized, but repeat CT revealed that the superior mesenteric artery dissection and intestinal wall thickening were unchanged. No intestinal ischemia was observed on computed tomography (CT). Esophagogastroduodenoscopy performed 13 days after angiography showed an improvement in the damaged duodenal mucosa (Figures 5(c) and 5(d)). CT performed the following day revealed improvement in mural thickening of the duodenum (Figure 4(f), arrow), jejunum, and ascending colon (Figure 4(f), arrowhead). The patient was started on liquid diet. Subsequently, the patient recovered uneventfully and was discharged 31 days after angiography.

## 3. Discussion

The prevalence of abdominal visceral artery aneurysms has been reported to be 0.01%–0.2% of the population [3]. With regard to visceral artery aneurysms, the splenic artery is the most often involved, accounting for 60%–80% of cases, followed by the hepatic artery, mesenteric artery, celiac artery, gastric and gastroepiploic arteries, gastroduodenal artery and its pancreatic branches, jejunal and ileocolic arteries, and inferior mesenteric artery [6]. Most visceral aneurysms occur in association with arterial media degeneration due to atherosclerosis, fibromuscular dysplasia, or collagen vascular disorders [7]. Among the presented patients, Case 1 had stenosis of the celiac artery with median arcuate ligament syndrome, and Case 2 had occlusion of the celiac artery. It is known that stenosis/occlusion of the celiac axis is responsible for aneurysm formation as a consequence of increased flow in the collateral vessels [8]. Therefore, the etiology in the present two cases was suspected to be stenosis/occlusion of the celiac artery. Although a size >2 cm is considered the threshold for surgical or endovascular treatment [9, 10], rupture of visceral aneurysms <2 cm has been reported. For instance, the risk of rupture of pancreaticoduodenal artery aneurysms with celiac stenosis/occlusion is reportedly independent of aneurysmal size and is associated with a 50% mortality rate [11].

Because it is less invasive than open surgery, endovascular embolization is the first-line therapy for abdominal visceral artery aneurysms [12]. In addition to aneurysm treatment, transcatheter embolization has been used for the management of gastrointestinal bleeding refractory to endoscopic hemostasis and transarterial chemoembolization of hepatocellular carcinoma. Adverse events related to endovascular treatment include bleeding, arterial dissection, intestinal ischemia, intestinal stenosis, coil migration [3, 13], pancreatitis [14], and access-site hematomas. Major ischemic complications of the gastrointestinal tract reportedly range from 0% to 16% [14, 15]. In Case 1, the proximal side of the aneurysm ruptured during the endovascular procedure. Although bleeding from the ruptured vessel was successfully managed with embolization using N-butyl cyanoacrylate, duodenal ulcer developed. It has been reported that the use of liquids, such as cyanoacrylates, or particulate embolic agents such as polyvinyl alcohol, is a risk factor for intestinal ischemic complications [14, 16].

In Case 2, iatrogenic dissection of the superior mesenteric artery occurred as an adverse event of an endovascular attempt to treat an aneurysm. Although the number of articles was limited, similar cases have been previously reported. Dzieciuchowicz et al. reported a case of a 42-year-old woman with pancreaticoduodenal artery aneurysms and stenosis of the celiac trunk [17]. Emergency laparotomy and thromboendarterectomy were performed, and magnetic resonance angiography performed 30 months after surgery showed patency of the superior mesenteric artery. In contrast, Tajima et al. conservatively treated a patient with superior mesenteric artery dissection that developed during angiography with peroral ticlopidine [18]. CT performed four weeks after angiography revealed patency of the lumen of the superior mesenteric artery.

We speculate that the mechanism of ischemia differed between Cases 1 and 2. In case 1, the arterial branches of the pancreaticoduodenal arcade were nonselectively clogged with N-butyl cyanoacrylate. In such patients, because vascular occlusion occurs in the peripheral parts of the artery, it is assumed that the degree of intestinal damage essentially depends on the extent of the embolic agent and the blood vessels that are affected. In contrast, in Case 2, duodenal ulceration developed due to a partial decrease in blood flow in all branches of the superior mesenteric artery, including the pancreaticoduodenal arcade and arteries supplying the small intestine and colon. In such cases, collateral circulation between the celiac and mesenteric arteries probably has protective effects against ischemic insults to the intestines, while there is a potential risk of deterioration of the bowel infarction as arterial dissection progresses. These differences are merely speculation, as there was no apparent hypertrophy of the arterial arcades or emergence of arteries to compensate for the decreased blood flow in Case 2. However, these differences in the mechanisms of ischemia can theoretically influence patient management and prognosis.

Careful observation is required in patients with endovascular treatment-related bowel ischemia because cases of bowel perforation developing after arterial embolization for pancreaticoduodenal artery aneurysm [19] and hepatocellular carcinoma [20] have been previously reported. In the two patients reported herein, duodenal ulcers occurred as an adverse event of transarterial treatment of unruptured abdominal visceral artery aneurysms. However, both patients recovered spontaneously with the administration of nothing by mouth, intravenous fluids, or proton-pump inhibitors. Antibiotics and heparin were administered in Case 2. Esophagogastroduodenoscopy was useful for evaluating the degree of duodenal mucosal damage at the onset and its chronological changes during the course of treatment. We consider that progressive deterioration or unresponsive mucosal damage during the course of treatment requires surgical management of the gastrointestinal tract, whereas conservative management is an option when improvement in mucosal damage is observed endoscopically. Thus, endoscopic examination is valuable in patients with endovascular treatment-related bowel ischemia, as it provides important information for optimal management. We recommend performing endoscopy as soon as possible, most desirably on the same day or the day after the ischemic event, to evaluate mucosal damage at onset. In Case 1, since endovascular treatment was performed on Friday, we performed endoscopy on Monday (3 days after the ischemic event), whereas we performed endoscopy the day after endovascular treatment in Case 2. Prior to performing endoscopy, it is mandatory to confirm the absence of signs of peritonitis on physical examination and CT. In addition, the use of carbon dioxide for insufflation is encouraged to decrease the risk of perforation [21].

## 4. Conclusions

We report two patients with duodenal ulcers that developed after transarterial embolization of unruptured abdominal visceral artery aneurysms. Both patients recovered spontaneously with conservative treatment. These cases highlight the usefulness of endoscopic observation in evaluating the degree of duodenal mucosal damage and supporting the decision-making process during conservative treatment of duodenal ulcers.

## Data Availability

The data that support the findings of this study are available from the corresponding author upon reasonable request.
